# 4-Pheneth­oxy­aniline hemihydrate

**DOI:** 10.1107/S1600536812022994

**Published:** 2012-05-26

**Authors:** Toheed Akhter, Humaira Masood Siddiqi, Zareen Akhter, Vickie McKee

**Affiliations:** a Department of Chemistry, Quaid-I-Azam University, Islamabad 45320, Pakistan; b Chemistry Department, Loughborough University, Loughborough LE11 3TU, England

## Abstract

The crystal structure of the title compound, C_14_H_15_NO·0.5H_2_O, features N—H⋯O and O—H⋯N hydrogen bonds between the amino group and water molecule of crystallization, which generate a chain along the *c* axis. The water mol­ecule lies on a twofold rotation axis. A C—H⋯π inter­action is observed between the phenyl and aniline rings. The angle between the mean planes of the phenyl rings is 72.51 (7)°.

## Related literature

For a similar hydrogen-bonding pattern in a related structure see Haider *et al.* (2011 ▶). The title compound is a precursor of diamine monomers, which are widely used for synthesis of polyimides (PIs), see: Ragosta *et al.* (2011 ▶). For the solubility of PIs, see: Chang *et al.* (2010 ▶). Reduced solubility is associated with inter­molecular hydrogen-bonding chains and stiffness, see: Hsiao & Leu (2004 ▶); Liaw *et al.* (2005 ▶). Incorporation of bulky pendant groups such as substituted phenyl groups into a rigid PI backbone can enhance the solubility of PIs, see: Liaw *et al.* (2005 ▶); Li *et al.* (2007 ▶). 
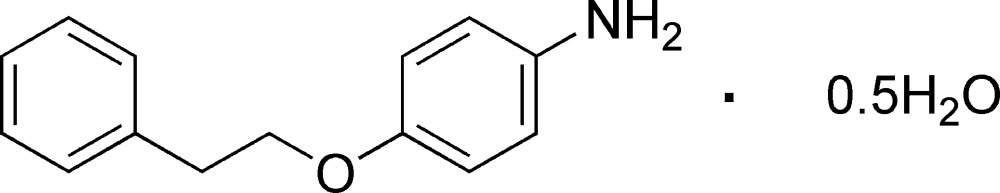


## Experimental

### 

#### Crystal data

C_14_H_15_NO·0.5H_2_O
*M* *_r_* = 222.28
Orthorhombic, 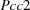

*a* = 11.6046 (8) Å
*b* = 13.1937 (10) Å
*c* = 7.9114 (6) Å
*V* = 1211.30 (15) Å^3^
*Z* = 4
Mo *K*α radiation
μ = 0.08 mm^−1^
*T* = 150 K
0.37 × 0.26 × 0.11 mm

#### Data collection

Bruker APEXII CCD diffractometer
Absorption correction: multi-scan (*SADABS*; Sheldrick, 2008 ▶) *T*_min_ = 0.971, *T*_max_ = 0.991
11920 measured reflections
1633 independent reflections
1443 reflections with *I* > 2σ(*I*)
*R*_int_ = 0.035

#### Refinement

*R*[*F* ^2^ > 2σ(*F* ^2^)] = 0.041
*wR*(*F* ^2^) = 0.115
*S* = 1.09
1633 reflections
159 parameters
6 restraints
H atoms treated by a mixture of independent and constrained refinement
Δρ_max_ = 0.20 e Å^−3^
Δρ_min_ = −0.31 e Å^−3^

### 

Data collection: *APEX2* (Bruker, 1998 ▶); cell refinement: *SAINT* (Bruker, 1998 ▶); data reduction: *SAINT*; program(s) used to solve structure: *SHELXS97* (Sheldrick, 2008 ▶); program(s) used to refine structure: *SHELXL97* (Sheldrick, 2008 ▶); molecular graphics: *SHELXTL* (Sheldrick, 2008 ▶); software used to prepare material for publication: *SHELXTL*.

## Supplementary Material

Crystal structure: contains datablock(s) I, global. DOI: 10.1107/S1600536812022994/kp2413sup1.cif

Structure factors: contains datablock(s) I. DOI: 10.1107/S1600536812022994/kp2413Isup2.hkl

Supplementary material file. DOI: 10.1107/S1600536812022994/kp2413Isup3.cml

Additional supplementary materials:  crystallographic information; 3D view; checkCIF report

## Figures and Tables

**Table 1:** Hydrogen-bond geometry (Å, °) *Cg* is the centroid of the C1–C6 ring.

*D*—H⋯*A*	*D*—H	H⋯*A*	*D*⋯*A*	*D*—H⋯*A*
N1—H1*A*⋯O1*W*^i^	0.83 (2)	2.20 (2)	3.011 (4)	166 (4)
O1*W*—H1*W*⋯N1	0.87 (2)	1.92 (2)	2.791 (4)	171 (3)
C14—H14⋯*Cg*^ii^	0.95	2.64	3.586 (3)	173

Symmetry codes: (i) 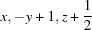; (ii) 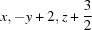.

**Fig. 1.**
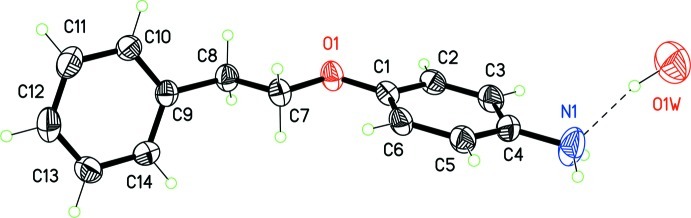
The view of the asymmetric unit (O1W lies on a 2-fold axis). H-bond shown as a dashed line and thermal ellipsoids shown at 50% probability.

**Fig. 2.**
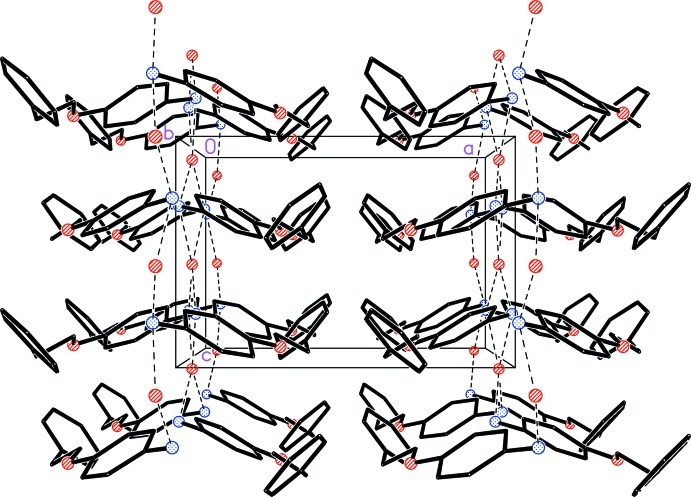
Packing diagram showing the H-bonded chains parallel to the c axis. H atoms omitted for clarity.

**Fig. 3.**
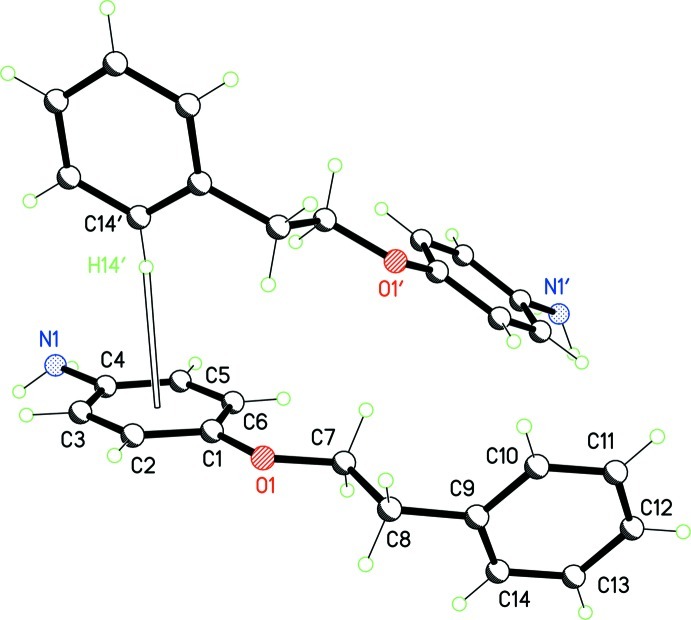
C—H ··· π interaction: C14···.Cg (C1-C6)is 3.586 (3) Å.

## supplementary crystallographic information

### Comment

The title compound is a precursor of diamine monomers which are widely used for synthesis of polyimides (PIs) (Ragosta *et al.*, 2011). Solubility of PIs is considered as one of the key property when they are used for their specific application (Chang *et al.*, 2010). Reduced solubility is associated with intermolecular hydrogen bonding chain and stiffness (Hsiao and Leu, 2004, Liaw *et al.*, 2005). Incorporation of bulky pendant groups such as substituted phenyl groups into rigid PI backbone can enhance the solubility of PIs (Liaw *et al.*, 2005, Li *et al.*, 2007). Synthesis of the title compound is aimed for introducing pendant substituted phenyl group into PI backbone and thereby improving their solubility.

Within the 1-amino-4-phenethoxybenzene molecule the two phenyl rings are tilted with respect to one another; the angle between the mean planes of the phenyl groups is 72.51 (7)° (Fig 1). The crystal structure is governed by hydrogen bonds between water molecule and the –NH_2_ group (Table 1, Fig. 2) generating hydrogen bonded chains running parallel to the *c* axis (Fig. 2). There is also a C—H···π interaction (Fig 3).

### Experimental

A mixture of 2-phenylethanol (5 g, 40.92 mmol), anhydrous potassium carbonate (5.68 g, 40.92 mmol) and dimethyl formamide (DMF, 60 mL) was stirred for 1 h in three-necked round bottom flask under the inert atmosphere of nitrogen. Afterward solution of 1-fluoro-4-nitrobenzene in DMF was added drop wise and the reaction mixture was refluxed for 24 h at 393 K. After complete consumption of reactants, light yellow colour product was precipitated by pouring the reaction mixture into distilled water (500 mL). The crude product, after filtration and washing thoroughly with distilled water, was recrystallised from possible minimum volume of absolute ethanol. Yield was 81%.

### Refinement

H atoms bonded to C atoms were inserted at calculated positions with C—H distances of 0.95 and 0.99 Å for aromatic and methylene C atoms, respectively. They were refined using a riding model with *U*_iso_(H) = 1.2*U*_eq_(C). H atoms bonded to O and N were located from difference maps and their coordinated refinded under geometric restraints, with *U*_iso_(H) = 1.5*U*_eq_(N or O).

### Crystal data

**Table:**

C_14_H_15_NO·0.5H_2_O	*F*(000) = 476
*M**_r_* = 222.28	*D*_x_ = 1.219 Mg m^−^^3^
Orthorhombic, *P**c**c*2	Mo *K*α radiation, λ = 0.71073 Å
Hall symbol: P 2 -2c	Cell parameters from 2822 reflections
*a* = 11.6046 (8) Å	θ = 3.1–24.1°
*b* = 13.1937 (10) Å	µ = 0.08 mm^−^^1^
*c* = 7.9114 (6) Å	*T* = 150 K
*V* = 1211.30 (15) Å^3^	Block, colourless
*Z* = 4	0.37 × 0.26 × 0.11 mm

### Data collection

**Table:**

Bruker APEXII CCD diffractometer	1633 independent reflections
Radiation source: fine-focus sealed tube	1443 reflections with *I* > 2σ(*I*)
Graphite monochromator	*R*_int_ = 0.035
ω rotation with narrow frames scans	θ_max_ = 28.3°, θ_min_ = 1.5°
Absorption correction: multi-scan (*SADABS*; Sheldrick, 2008)	*h* = −15→15
*T*_min_ = 0.971, *T*_max_ = 0.991	*k* = −17→17
11920 measured reflections	*l* = −10→10

### Refinement

**Table:**

Refinement on *F*^2^	Primary atom site location: structure-invariant direct methods
Least-squares matrix: full	Secondary atom site location: difference Fourier map
*R*[*F*^2^ > 2σ(*F*^2^)] = 0.041	Hydrogen site location: inferred from neighbouring sites
*wR*(*F*^2^) = 0.115	H atoms treated by a mixture of independent and constrained refinement
*S* = 1.09	*w* = 1/[σ^2^(*F*_o_^2^) + (0.0593*P*)^2^ + 0.2388*P*] where *P* = (*F*_o_^2^ + 2*F*_c_^2^)/3
1633 reflections	(Δ/σ)_max_ < 0.001
159 parameters	Δρ_max_ = 0.20 e Å^−^^3^
6 restraints	Δρ_min_ = −0.31 e Å^−^^3^

### Special details

**Table:**

Geometry. All e.s.d.'s (except the e.s.d. in the dihedral angle between two l.s. planes) are estimated using the full covariance matrix. The cell e.s.d.'s are taken into account individually in the estimation of e.s.d.'s in distances, angles and torsion angles; correlations between e.s.d.'s in cell parameters are only used when they are defined by crystal symmetry. An approximate (isotropic) treatment of cell e.s.d.'s is used for estimating e.s.d.'s involving l.s. planes.
Refinement. Refinement of *F*^2^ against ALL reflections. The weighted *R*-factor *wR* and goodness of fit *S* are based on *F*^2^, conventional *R*-factors *R* are based on *F*, with *F* set to zero for negative *F*^2^. The threshold expression of *F*^2^ > σ(*F*^2^) is used only for calculating *R*-factors(gt) *etc*. and is not relevant to the choice of reflections for refinement. *R*-factors based on *F*^2^ are statistically about twice as large as those based on *F*, and *R*- factors based on ALL data will be even larger.Freidel equivalents merged since the data do not permit determination of absolute configuration.

### Fractional atomic coordinates and isotropic or equivalent isotropic displacement parameters (Å^2^)

**Table:**

	*x*	*y*	*z*	*U*_iso_*/*U*_eq_	Occ. (<1)
O1	0.69069 (13)	0.95389 (11)	0.9053 (2)	0.0359 (4)	
C1	0.77654 (19)	0.88571 (15)	0.8679 (3)	0.0300 (4)	
C2	0.7603 (2)	0.78822 (16)	0.9316 (3)	0.0335 (5)	
H2	0.6926	0.7727	0.9940	0.040*	
C3	0.8422 (2)	0.71407 (16)	0.9041 (3)	0.0371 (5)	
H3	0.8301	0.6478	0.9477	0.044*	
C4	0.94237 (19)	0.73527 (16)	0.8132 (3)	0.0367 (5)	
N1	1.0264 (2)	0.65890 (16)	0.7857 (5)	0.0583 (8)	
H1B	1.091 (2)	0.680 (3)	0.766 (5)	0.087*	
H1A	1.024 (3)	0.623 (3)	0.872 (4)	0.087*	
C5	0.95608 (19)	0.83219 (15)	0.7463 (4)	0.0350 (5)	
H5	1.0226	0.8473	0.6810	0.042*	
C6	0.87417 (18)	0.90678 (15)	0.7735 (3)	0.0321 (5)	
H6	0.8850	0.9726	0.7273	0.039*	
C7	0.70920 (19)	1.05805 (15)	0.8599 (3)	0.0313 (5)	
H7A	0.7072	1.0661	0.7355	0.038*	
H7B	0.7851	1.0815	0.9016	0.038*	
C8	0.61305 (18)	1.11875 (16)	0.9413 (3)	0.0340 (5)	
H8A	0.5377	1.0935	0.9003	0.041*	
H8B	0.6156	1.1093	1.0654	0.041*	
C9	0.62423 (18)	1.23026 (15)	0.9003 (3)	0.0290 (4)	
C10	0.5572 (2)	1.27416 (17)	0.7746 (3)	0.0354 (5)	
H10	0.5018	1.2342	0.7165	0.042*	
C11	0.5704 (2)	1.37604 (18)	0.7328 (3)	0.0421 (6)	
H11	0.5251	1.4049	0.6451	0.051*	
C12	0.6490 (2)	1.43509 (18)	0.8186 (4)	0.0423 (6)	
H12	0.6577	1.5047	0.7905	0.051*	
C13	0.71508 (19)	1.39267 (18)	0.9455 (4)	0.0408 (6)	
H13	0.7687	1.4334	1.0055	0.049*	
C14	0.70341 (19)	1.29072 (18)	0.9856 (3)	0.0346 (5)	
H14	0.7499	1.2620	1.0720	0.042*	
O1W	1.0000	0.5000	0.5561 (6)	0.0725 (10)	
H1W	1.005 (5)	0.5538 (8)	0.620 (4)	0.109*	
X1A	0.8586	0.8104	0.8394	0.040*	0.00

### Atomic displacement parameters (Å^2^)

**Table:**

	*U*^11^	*U*^22^	*U*^33^	*U*^12^	*U*^13^	*U*^23^
O1	0.0365 (8)	0.0254 (7)	0.0457 (10)	−0.0005 (6)	0.0102 (8)	0.0010 (7)
C1	0.0334 (10)	0.0252 (9)	0.0313 (10)	−0.0025 (8)	0.0001 (9)	−0.0028 (8)
C2	0.0362 (11)	0.0304 (11)	0.0338 (12)	−0.0068 (8)	0.0013 (10)	0.0018 (9)
C3	0.0451 (12)	0.0250 (10)	0.0411 (12)	−0.0050 (9)	−0.0031 (11)	0.0062 (9)
C4	0.0345 (11)	0.0266 (10)	0.0490 (14)	0.0016 (8)	−0.0050 (11)	−0.0002 (10)
N1	0.0451 (13)	0.0311 (10)	0.099 (2)	0.0077 (9)	0.0094 (15)	0.0114 (13)
C5	0.0325 (10)	0.0277 (10)	0.0448 (13)	−0.0040 (8)	0.0026 (10)	−0.0010 (10)
C6	0.0359 (11)	0.0228 (9)	0.0377 (11)	−0.0032 (8)	0.0038 (10)	−0.0001 (9)
C7	0.0354 (10)	0.0251 (9)	0.0334 (11)	0.0000 (8)	0.0044 (9)	0.0000 (8)
C8	0.0325 (10)	0.0294 (10)	0.0400 (13)	0.0011 (8)	0.0055 (10)	−0.0002 (9)
C9	0.0282 (9)	0.0291 (9)	0.0296 (10)	0.0041 (8)	0.0058 (8)	−0.0020 (9)
C10	0.0358 (11)	0.0363 (11)	0.0340 (12)	0.0072 (9)	−0.0064 (10)	−0.0084 (9)
C11	0.0477 (14)	0.0405 (12)	0.0382 (13)	0.0155 (11)	−0.0042 (11)	0.0002 (11)
C12	0.0456 (13)	0.0281 (10)	0.0532 (16)	0.0072 (9)	0.0074 (12)	0.0018 (11)
C13	0.0329 (11)	0.0350 (11)	0.0546 (16)	−0.0039 (9)	−0.0013 (11)	−0.0069 (11)
C14	0.0298 (10)	0.0376 (11)	0.0364 (12)	0.0005 (9)	−0.0043 (9)	0.0003 (10)
O1W	0.083 (2)	0.0438 (14)	0.090 (3)	−0.004 (2)	0.000	0.000

### Geometric parameters (Å, º)

**Table:**

O1—C1	1.374 (3)	C8—C9	1.512 (3)
O1—C7	1.437 (2)	C8—H8A	0.9900
C1—C6	1.385 (3)	C8—H8B	0.9900
C1—C2	1.394 (3)	C9—C10	1.389 (3)
C2—C3	1.381 (3)	C9—C14	1.391 (3)
C2—H2	0.9500	C10—C11	1.393 (3)
C3—C4	1.396 (3)	C10—H10	0.9500
C3—H3	0.9500	C11—C12	1.378 (4)
C4—C5	1.393 (3)	C11—H11	0.9500
C4—N1	1.419 (3)	C12—C13	1.382 (4)
N1—H1B	0.818 (18)	C12—H12	0.9500
N1—H1A	0.834 (18)	C13—C14	1.389 (3)
C5—C6	1.385 (3)	C13—H13	0.9500
C5—H5	0.9500	C14—H14	0.9500
C6—H6	0.9500	O1W—H1W	0.873 (19)
C7—C8	1.517 (3)	X1A—H14^i^	2.6415
C7—H7A	0.9900	X1A—C14^i^	3.586 (2)
C7—H7B	0.9900		
			
C1—O1—C7	117.64 (17)	H7A—C7—H7B	108.6
O1—C1—C6	125.28 (18)	C9—C8—C7	111.07 (17)
O1—C1—C2	115.34 (19)	C9—C8—H8A	109.4
C6—C1—C2	119.4 (2)	C7—C8—H8A	109.4
C3—C2—C1	120.2 (2)	C9—C8—H8B	109.4
C3—C2—H2	119.9	C7—C8—H8B	109.4
C1—C2—H2	119.9	H8A—C8—H8B	108.0
C2—C3—C4	120.8 (2)	C10—C9—C14	118.5 (2)
C2—C3—H3	119.6	C10—C9—C8	120.7 (2)
C4—C3—H3	119.6	C14—C9—C8	120.7 (2)
C5—C4—C3	118.4 (2)	C9—C10—C11	120.8 (2)
C5—C4—N1	121.0 (2)	C9—C10—H10	119.6
C3—C4—N1	120.6 (2)	C11—C10—H10	119.6
C4—N1—H1B	115 (3)	C12—C11—C10	120.1 (2)
C4—N1—H1A	104 (3)	C12—C11—H11	120.0
H1B—N1—H1A	112 (3)	C10—C11—H11	120.0
C6—C5—C4	121.0 (2)	C11—C12—C13	119.7 (2)
C6—C5—H5	119.5	C11—C12—H12	120.1
C4—C5—H5	119.5	C13—C12—H12	120.1
C1—C6—C5	120.2 (2)	C12—C13—C14	120.3 (2)
C1—C6—H6	119.9	C12—C13—H13	119.9
C5—C6—H6	119.9	C14—C13—H13	119.9
O1—C7—C8	106.79 (17)	C13—C14—C9	120.6 (2)
O1—C7—H7A	110.4	C13—C14—H14	119.7
C8—C7—H7A	110.4	C9—C14—H14	119.7
O1—C7—H7B	110.4	H14^i^—X1A—C14^i^	1.9
C8—C7—H7B	110.4		

Symmetry code: (i) *x*, −*y*+2, *z*−1/2.

### Hydrogen-bond geometry (Å, º)

Cg is the centroid of the C1–C6 ring.

**Table:**

*D*—H···*A*	*D*—H	H···*A*	*D*···*A*	*D*—H···*A*
N1—H1*A*···O1*W*^ii^	0.83 (2)	2.20 (2)	3.011 (4)	166 (4)
O1*W*—H1*W*···N1	0.87 (2)	1.92 (2)	2.791 (4)	171 (3)
C14—H14···*Cg*^iii^	0.95	2.64	3.586 (3)	173

Symmetry codes: (ii) *x*, −*y*+1, *z*+1/2; (iii) *x*, −*y*+2, *z*+3/2.
